# Social support and psychological well-being in younger and older adults: The mediating effects of basic psychological need satisfaction

**DOI:** 10.3389/fpsyg.2022.1051968

**Published:** 2022-11-25

**Authors:** Huiyoung Shin, Chaerim Park

**Affiliations:** Department of Psychology, Jeonbuk National University, Jeonju, South Korea

**Keywords:** social support, basic psychological need, happiness, depressive symptoms, age-group differences

## Abstract

This study examined the associations between social support from different relationship types (i.e., spouses, children, friends, and parents) and psychological well-being (i.e., happiness and depressive symptoms), and the mediating effects of basic psychological need satisfaction (i.e., autonomy, competence, and relatedness) in these associations. A dataset on social support, satisfaction of basic psychological needs, and psychological well-being was collected from 823 South Korean adults (the mean of age = 48.83; 50.40% male). Results showed that social support from spouses and friends had the most robust associations with happiness and depressive symptoms. In addition, the satisfaction of needs for autonomy and relatedness mediated the associations between social support from spouses and friends, and happiness and depressive symptoms. However, these associations differed by age groups. Although social support satisfied basic psychological needs better for younger adults than for older adults, the satisfaction of needs for autonomy and relatedness was critical in order to support well-being for both groups.

## Introduction

The beneficial effects of social support on psychological well-being and health have been established over decades of research (Holt-Lunstad et al., 2010; Saphire-Bernstein and Taylor, 2013). Research has consistently shown that individuals with close and supportive spouses, friends, and family have greater life satisfaction and well-being (Antonucci and Jackson, 1987; Chen and Feeley, 2014) and fewer psychological and health-related costs, such as loneliness, depressive symptoms, and cognitive deficit (Okabayashi et al., 2004; Sherman et al., 2011). On the other hand, lack of social support has been implicated in emotional distress, depressive symptoms, and morbidity (Yang et al., 2014; Lerman Ginzburg et al., 2021).

Within the literature, diverse theoretical frameworks have been presented to understand the processes, such as attachment, belonging, intimacy, and social integration, whereby supportive relationships affect psychological well-being (Ainsworth et al., 1978; Baumeister and Leary, 1995; Berkman et al., 2000; Seeman, 2001). Researchers have also vigorously examined a wide range of variables that attest to the importance of social support for individuals’ well-being and optimal psychological functioning (Cohen and Wills, 1985; Folkman and Lazarus, 1986; see Lincoln, 2000 for a review). Several theoretical models and empirical research have characterized the diverse ways in which positive relationships contribute to psychological well-being.

Based on self-determination theory (SDT; Deci and Ryan, 1985), which posits that the satisfaction of the three basic psychological needs is necessary for individuals’ well-being and thriving, the overall aim of this study was to examine whether younger and older adults’ perceived social support from different relationship types would relate to their psychological well-being *via* satisfaction of basic psychological needs. Although prior research has examined whether the satisfaction of basic psychological needs acts as explanatory mechanism in the associations between social support and psychological well-being, most studies have often aggregated the different needs for autonomy, competency, and relatedness into a global measure and have considered overall satisfaction of needs without differentiating the unique effects of each specific need. Thus, it is difficult to assess the relative importance of different psychological needs for psychological well-being (Abidin et al., 2022; Vermote et al., 2022).

Furthermore, many studies investigating the link between social support and well-being have used groups of participants who are developmentally homogeneous, such as adolescents, young adults (Martela and Ryan, 2016; Tian et al., 2016) or older adults (Neubauer et al., 2017; Chen and Zhang, 2021). Thus, it is not clear if there are differences between age groups in the importance and impact of social support and satisfaction of basic psychological needs on psychological well-being. Although social support has pervasive benefits throughout the adult lifespan, the relative salience of different relationships and the satisfaction of needs could change over time due to age-related losses in behavioral and psychological functioning and social circumstances associated with age (Baltes and Baltes, 1990; Carstensen, 2006).

Thus, to elucidate the associations between social support, satisfaction of basic psychological needs, and well-being, we examined the relative importance of satisfaction of each specific need (i.e., for autonomy, competence, and relatedness) in mediating roles, and investigated the potential differences between younger (30–59 years) and older adults (60 years and older). We compared such two groups because many prior studies have considered adults aged 60 and over as older adults (Okabayashi et al., 2004; Giasson et al., 2017), and the official retirement age is 60 in South Korea. Retirement is a significant life event which characterizes the transition to a new life phase (Henning et al., 2016). As retirees withdraw from work, they have more free time available to use, but they also experience shrinking social networks associated with retirement. Diminished roles and expectations of retirees could shape the nature of their social interactions differently compared with non-retired younger adults. There have not yet been studies that examined whether there are differences by age groups in the associations between social support, satisfaction of needs, and psychological well-being. With our research design, this study could afford a unique information about if social support from certain relationships becomes more important for satisfaction of needs, and whether the satisfaction of certain needs becomes increasingly more important for psychological well-being as people age.

### Conceptualization of social support

How social support is defined and assessed include various aspects of support. For example, we can define and measure actual received support, perceived availability of support, frequency of social interactions, and density of social networks. However, definitions of most studies include the provision of support that can range from emotional to instrumental (Antonucci and Jackson, 1987). Emotional support is perceived notion that a person is esteemed and accepted. By communicating to individuals that they are valued and accepted despite any faults, individuals’ self-esteem can be strengthened; so, it is also referred to as esteem support (Cobb, 1976). Informational support refers to help in understanding and coping with challenges; so, it is also referred to as cognitive guidance or appraisal support. Instrumental support involves the provision of tangible support such as financial help and needed services (Cohen and Wills, 1985). Although features and functions of social support can be differentiated conceptually, they are not generally independent in real life. Those who display caring and understanding are also likely to offer informational or instrumental support. In the present study, we operationalize social support as one’s perceived notion of the caring and understanding. Also, although we did not differentiate its functions, we focused on aspects of emotional and informational support as they are most likely to be responsive to a wide range of stressful events.

### Social support as a main effect and a stress buffer

Research based on the main effect model indicates that regular social interactions with diverse relationship partners provide individuals positive affect, a sense of stability in life, and a recognition of self-worth, all of which positively predict overall psychological well-being (Thoits, 1983; Cohen et al., 2000). Researchers have also provided evidence for buffering effects such that social support can lessen costs of stressful events on psychological well-being (the stress-buffering effect model; Cohen and Wills, 1985). The stress-buffering effect model focuses on individuals’ appraisal of stressful events and coping responses (Folkman and Lazarus, 1986) and underscores that social support plays a role in the causal chain linking stressful events to illness or health-related behaviors (Cohen and McKay, 1984). That is, perceived (or availability) of social support can redefine the potential for harm induced by stressful events or bolster individuals’ capabilities to cope with imposed stresses, and thus preventing a stress appraisal response. Social support can also alleviate the impact of stressful events by providing solutions to the problems or re-evaluating the significance of the problems, so that individuals can be less responsive to stresses (House, 1981). Considerable evidence supports these main effect and stress-buffering effect models showing that greater social support promotes happiness and psychological well-being (Antonucci et al., 2013; Secor et al., 2017) and alleviates loneliness and depressive symptoms (Seeman et al., 1995; Chen and Feeley, 2014).

### Social support from different relationship types and psychological well-being

Individuals are surrounded by diverse social interactions partners, and each relationship can have distinct implications for their well-being (Rook et al., 1991; Fingerman, 1996; Carr et al., 2020). According to the convoy model (Antonucci, 2001), roles and demands are one of the critical situational factors that influence the features of individuals’ social relationships. Evidence indicates that having multiple social roles promotes social integration and connectedness and thus, improve individuals’ well-being (Moen, 2001). Similar to the convoy model, theory of the functional specificity of relationships (Weiss, 1974) also suggests that different social relationships provide various functional benefits for individuals, including attachment (e.g., support by spouses), reliable alliance (e.g., social support by family), reassurance of self-worth (e.g., support by friends), and opportunity for nurturance (e.g., support by children). Thus, it can be expected that social support occurring across multiple relationships can have additive benefits for individuals’ psychological well-being.

Indeed, prior research that simultaneously considered individuals’ different social relationships based on the pattern-centered approach demonstrated that various relationship profiles are differentially linked to individuals’ well-being (Fiori et al., 2006; Birditt and Antonucci, 2007). These findings provided a broader perspective on the association between multiple relationships and well-being. For example, findings showed that having multiple high quality social relationships, not necessarily with a spouse, is associated with greater psychological well-being. However, if individuals do not have close friends and their support, spousal support was particularly critical for their well-being. In a similar vein, a few studies differentiated the types of relationships and examined if social support by spouses, family, and friends has different effects on psychological well-being (Okabayashi et al., 2004; Chen and Feeley, 2014). These findings indicated that social support from different relationship types exhibit distinct influence on positive and negative aspects of psychological well-being, but spousal support has the greatest effects on well-being among all possible relationships. Although these studies demonstrated differential but additive influence of multiple relationships on well-being, because most of them have focused on adults aged 50 or 60 years and older, the evidence is not sufficient to fully understand the similarities and differences in the associations between social support from different relationship types and well-being between varying age groups. Thus, we build on this line of inquiry by examining social support received from spouses, children, parents, and friends in relation to happiness and depressive symptoms using younger and older adults.

### The mediating effects of satisfaction of basic psychological needs

Self-determination theory (SDT) proposes that people function most effectively when fundamental psychological needs for autonomy, competence, and relatedness are satisfied by means of social-environmental support (Deci and Ryan, 2000). The need for autonomy indicates the inclination of individuals to have feelings of volition, willingness, and choice about their behaviors and actions. The need for competence concerns having a sense of self-efficacy and mastery in interactions with the social environment and feeling confident and effective about various tasks and aims. The need for relatedness refers to the inclination of individuals to have feelings of connectedness to significant others and to experience a sense of belonging in their relationships and social groups (Ryan and Deci, 2002).

Because supportive interaction partners provide individuals with caring and understanding, help in coping with challenges, and tangible support, social support should facilitate satisfaction of needs for autonomy, competence, and relatedness. For example, parents’ instrumental support in the form of financial or tangible resources could motivate individuals to have feelings of choice about behaviors and take self-directed action (i.e., autonomy), and spouses’ informational support in the form of help in coping with challenges could promote individuals’ self-efficacy and confidence about various tasks (i.e., competence; Cutrona and Suhr, 1992). Also, friends’ understanding should allow individuals to have feelings of connectedness and a sense of belonging (i.e., relatedness; Blieszner, 2014).

In addition, satisfaction of basic psychological needs is essential for individuals’ psychological well-being (Deci and Ryan, 2014). Hence the satisfaction of these three basic psychological needs contributes to individuals’ well-being and thriving, whereas the frustration of these needs increases psychological and health-related costs. Consistent with the SDT, prior research has established a link between satisfaction of needs and psychological well-being. When individuals’ basic psychological needs were satisfied, that contributed to their life satisfaction (Tay and Diener, 2011), vitality (Deci and Ryan, 2008), and longevity (Weinstein et al., 2019). In contrast, having psychological needs be thwarted was related to depressive symptoms (Vandenkerckhove et al., 2020) and greater stress (Reeve and Tseng, 2011).

Thus, satisfaction of basic psychological needs could mediate the link between social support and psychological well-being. Indeed, there are several recent research that has shown that the satisfaction of needs mediates the associations between social support and various indicators of well-being (Vermote et al., 2022). However, these studies have considered overall satisfaction of needs without distinguishing the separate measured needs. Because SDT emphasizes that the three needs are relatively independent and that each specific need could have distinct effects on individuals’ well-being (Ryan and Deci, 2017), considering overall satisfaction of needs would not strictly be compatible with the proposition of SDT (Dysvik et al., 2013) and may obscure the independent or additive effects of individual needs on well-being.

Although the three needs are intercorrelated, each specific need is independently significant and has its own satisfaction criteria (Karkkola et al., 2018). In addition to different levels of fulfilment, if any of the three needs is thwarted or unmet, negative psychological repercussions could ensue (Ryan and Deci, 2017). Also, the three needs are mutually implicated and complementary in nature. Although the satisfaction of relatedness can have positive effects on well-being indicators, feeling relatedness alone does not fully ensure greater well-being. Flourishing relationship satisfaction and optimal psychological functioning also require fulfilment of the need for autonomy and competence (Patrick et al., 2007). Therefore, to better understand the relative importance and potential additive effects of the three needs on well-being, we distinguished the three individual needs and investigated whether satisfaction of each specific need for autonomy, competence, and relatedness, respectively, mediates the link between social support and psychological well-being.

### Age-group difference in the link between social support and well-being

As individuals make a transition across life stages, salient focal relationships evolve over time (Kahn and Antonucci, 1980) because personal and situational factors differently affect their relationships (Fiori et al., 2006). Accordingly, social support from multiple relationships is differentially valued and differ in its influence on well-being over adult life span (Carstensen, 1998). For example, younger and older adults do not have similar levels of desires for social support from their relationship partners (Antonucci and Akiyama, 1995). That is, social support that is effective at reducing loneliness at an earlier point in life would not be as important at a later point in life. In young adulthood, close friends are the major source of social support, with support from friends being more strongly related to psychological functioning than support from family (Allen et al., 2000). When individuals progress through middle and old age, social support from spouses and family members becomes more critical (Carstensen, 1995).

Life-span theorists suggest that older adults reap more benefits from social support from spouse and family than younger adults do because compared to younger adults who perceive unlimited time ahead of them, older adults would envision less amount of time left to live and would thus focus on those who are the most important to them (e.g., Carstensen, 1998). Thus, support from direct family members, such as spouses, becomes more critically tied to their well-being (Segrin, 2003). In addition, older adults generally experience a shrinkage of social networks because of retirement, disability, and relocation. Thus, older adults are more likely to compensate for diminishing social networks by adjusting their desires for support to a lower level, which would be more easily fulfilled (Rook, 2000). By doing so, older adults could preserve enhanced personal well-being despite declines in their social networks that might otherwise negatively affect their sense of well-being.

However, research findings about these associations are mixed. Some studies have demonstrated that family support remains important in young adults’ psychological functioning (Mounts et al., 2006), with friend support having more robust influence on older adults’ well-being than family support (Huxhold et al., 2014). They suggest that friend support has significant influence on well-being in later life because older adults usually confide in friends as their major sources of support related to the aging process as familial support naturally decreases over time due to death of a spouse and direct family members. Other studies have also indicated that social support from either friends or family is not related to well-being (e.g., loneliness and life satisfaction) among older adults (Mullins et al., 1996; McCamish-Svensson et al., 1999). Given this mixed evidence, empirical research to investigate such age-group differences is warranted.

### Age-group difference in the link between satisfaction of needs and well-being

Many studies have documented the beneficial effects of satisfaction of needs for individuals’ well-being for adolescents and young adults (see Ryan and Deci, 2017 for a review). Increasing research has also demonstrated the importance of satisfaction of needs for well-being among older adults (Henning et al., 2019). However, compared to the ample evidence for adolescents and young adults, only a handful of studies have considered the satisfaction of needs in relation to older adults’ psychological well-being. Also, little is known about age-group differences in the link between satisfaction of needs and well-being over the adult lifespan. Studies have usually been focused on individual differences using homogeneous groups of participants (e.g., Martela and Ryan, 2016; Neubauer et al., 2017), and have rarely investigated the satisfaction of specific needs and their mediating effects between social support and well-being for different age groups. Therefore, the literature concerning age-group differences in the associations between satisfaction of needs and well-being is small and equivocal.

It can be assumed that there could be a shift in the relative importance of satisfaction of specific needs as people age (Ryff, 1989), and psychological well-being of older adults might not be depended as much on the satisfaction of the same needs as in earlier life stages (Neubauer et al., 2017). Socioemotional selectivity theory (SST) suggests that when individuals perceive their time as limited, they value emotionally meaningful goals over goals directed toward gaining information (Carstensen, 2006). That is, the association between the satisfaction of relatedness and well-being could be particularly prominent among older adults. In addition, selective optimization with compensation (SOC) model indicates that aging requires individuals to select tasks that they can perform well in the processes of age-related losses in behavioral and psychological functioning, and such a narrowing down in selection of tasks can lead to optimization, allowing individuals to maintain their finest performance in areas that they prioritize and compensate for other tasks that they can no longer perform well (Baltes and Baltes, 1990). Hence efficient compensation would support optimization processes as people age and the satisfaction of the need for competence could be less critical for older adults’ well-being.

Lastly, satisfaction of the need for autonomy could have more importance for and more significant effects on well-being in the life of older adults. A few empirical studies support that satisfaction of the need for autonomy is associated with greater life satisfaction among older adults. For example, the satisfaction of the needs for autonomy and relatedness, but not competence, predicted subjective well-being among older adults aged between 60 and 66 years (Henning et al., 2019). In addition, the frustration of the need for autonomy was associated with negative affect among the elderly aged over 87 years (Neubauer et al., 2017). Findings from these studies are promising, in that they showed that the beneficial effects of satisfying the need for autonomy on well-being found in younger adults (Vansteenkiste et al., 2006) also applies for older adults. However, these studies did not consider younger and older adults simultaneously; so, it is not clear whether the effect of satisfying the need for autonomy on well-being is greater for older adults than for younger adults. Thus, we address this issue and examine the potential age-group differences in the link between satisfaction of needs and well-being.

### The current study

The aim of this study was to examine the associations between social support from different relationship types and psychological well-being, and the mediating effects of satisfaction of basic psychological needs in these associations using a large dataset of younger and older adults in South Korea. To address this aim, we focused on social support received from spouses, children, friends, and parents and two psychological well-being indicators, including happiness and depressive symptoms. To elucidate the relative importance of satisfaction of needs in their mediating effects, we simultaneously considered individual needs for autonomy, competence, and relatedness. To enrich understanding on the potential age-group differences, we used both younger (30–59 years) and older adults (60 years and older).

Based on the aforementioned theories and empirical evidence, we formulated three hypotheses. First, we hypothesized that social support received from spouses, children, friends, and parents could have different effects on happiness and depressive symptoms, with spousal support being most robustly associated with greater happiness and reduced depressive symptoms (Okabayashi et al., 2004; Birditt and Antonucci, 2007). Second, we hypothesized that the satisfaction of needs for autonomy, competence, and relatedness could mediate the associations between social support and well-being, with satisfaction of each need having distinctive effects for happiness and depressive symptoms (Karkkola et al., 2018; Chen and Zhang, 2021). Third, we hypothesized that the associations between social support, satisfaction of needs, and happiness and depressive symptoms could differ between younger and older adults. In particular, we anticipated that social support from spouses and family could have stronger effects on satisfaction of needs, and happiness and depressive symptoms for older adults than for younger adults (Carstensen, 1995). Similarly, we expected that the satisfaction of the needs for autonomy and relatedness could have stronger effects on happiness and depressive symptoms for older adults than for younger adults (Baltes and Baltes, 1990; Carstensen, 2006), whereas the satisfaction of the need for competence could have stronger effects on happiness and depressive symptoms for younger adults than for older adults (Neubauer et al., 2017; Henning et al., 2019).

## Materials and methods

### Participants

We collected data from an online research participant system after obtaining an ethical approval from the University’s Institutional Review Board in 2021.^1^ All of the participants provided their informed consent prior to starting the survey. The current study was based on 807 South Korean adults (50.40% male) aged between 30 and 69 years (the mean of age = 48.83, *SD* = 11.02; 30–39 years, *n* = 203; 40–49 years, *n* = 204; 50–59 years, *n* = 209; 60–69 years, *n* = 207). We collected a dataset by groups by decades and used two different age-groups of younger (i.e., between 30 and 59 years) and older adults (i.e., 60 years old and over). We provided detailed demographic information in the Appendix.

### Measures

#### Social support

We assessed social support from different relationships using positive and negative social support scales (Smith et al., 2013). It consists of 12 items measuring perceived social support for the four relationships. The scale consists of social support and social strain for the four different relationships, and we used the social support dimension in this study. Smith et al. (2013) reported the Cronbach’s αs to be 0.82, 0.83, 0.84, and 0.86 for social support for subscales of spouse, children, friends, and parents, respectively. Participants responded to each statement using a 5-point scale (1 = *not at all true* and 5 = *very true*). A sample item was, “How much can you rely on them if you have a serious problem?” The average score was calculated for each subscale, with higher scores indicating greater social support. The scores for Cronbach’s αs in this study were 0.86, 0.82, 0.84, and 0.83 for spouse, friends, parents, and children, respectively.

#### Satisfaction of basic psychological needs

The satisfaction of basic psychological needs was measured with the Basic Psychological Need Satisfaction Scale-general version (Gagné, 2003). The scale consists of 21 items assessing the satisfaction of each need (7 items for autonomy, 6 items for competence, and 8 items for relatedness). Gagné (2003) reported Cronbach’s α of 0.69, 0.71, and 0.86 for satisfaction of autonomy, competence, and relatedness, respectively. Participants responded to each statement using a 5-point scale (1 = *not at all true* and 5 = *very true*). Sample items included, “I feel like I am free to decide for myself how to live my life” for measuring autonomy, “I feel a sense of accomplishment from what I do” for measuring competence, and “People I interact with on a daily basis tend to take my feelings into consideration” for measuring relatedness. The average score was calculated for each subscale, with higher scores indicating greater satisfaction for each need. The Cronbach’s αs for this scale in this study were 0.80, 0.91, and 0.84 for autonomy, competence, and relatedness, respectively.

#### Happiness

Happiness was measured with the Oxford Happiness Questionnaire (OHQ) developed by Hills and Argyle (2002). The scale consists of 29 items assessing the levels of happiness represented by positive mood. Hills and Argyle (2002) reported a Cronbach’s α of 0.91. Participants reported to what extent they felt the way explained in each statement using a 5-point scale (1 = *not at all true* and 5 = *very true*). Sample statements included “I feel I have a great deal of energy.” and “I often experience joy and elation” The average score was calculated, with higher scores indicating greater happiness. This scale’s Cronbach’s α for this study was 0.92.

#### Depressive symptoms

Depressive symptoms were measured with the Center for Epidemiological Studies-Depressive symptoms (CES-D) Scale (Radloff, 1977). The scale consists of 20 items assessing depressed mood in a non-clinical sample of various ages. Radloff (1977) reported a Cronbach’s α of 0.85 for this scale. Participants reported how often during the past week they had experienced symptoms explained in the statements. Sample statements included “I felt I could not shake off the blues” and “I talked less than usual.” Each Item was scored from 0 (*rarely*) *to* 3 (*most or the time*), with higher scores indicating worse depressive symptoms. The Cronbach’s α of this scale was 0.94 for this study.

### Analytic strategy

All statistical analyses were conducted using R 4.1.0 and SPSS 25.0. As a preliminary analysis, descriptive statistics and Pearson correlations among the variables were computed. To investigate differences by age groups, we performed independent *t-*tests for all variables. To construct an integrated model where the associations between all constructs are simultaneously estimated, we incorporated structural equational modeling (SEM) using R 4.1.0 with the lavaan package (Rosseel, 2012). We followed the model fit criteria established by Byrne (2006): *χ*^2^/*df* ≤ 5.0; CFI and TLI ≥ 0.90; RMSEA and SRMR ≤0.08. Also, we used changes in CFI (∆CFI) as a guideline to compare the nested models: a change smaller than or equal to 0.01 indicates that the null hypothesis should not be rejected (Cheung and Rensvold, 2002). Before the main path analyses, we conducted confirmatory factor analysis to examine whether the measurement model provides a good fit to the data. We found that all of the constructs indicated acceptable and good fit indices (*χ*^2^/*df* = 3.25, *p* < 0.001, CFI = 0.95, TLI = 0.94, RMSEA = 0.05 (90% CI of the RMSEA = 0.05–0.06), and SRMR = 0.06), and all of the indicators loaded significantly on their respective constructs. Next, we investigated measurement invariance between younger and older adults by conducting multi-group confirmatory factor analysis (Byrne, 2010).^2^ We investigated the three nested models using maximum-likelihood estimation: (a) configural, (b) metric, and (c) scalar invariance, with the latter model indicating more stringent constraints (Byrne, 2010). Table 1 shows that each level of invariance model had a satisfactory fit, and there was no substantial decrease in model fit over that of the less-constrained models. Thus, we proceeded to our main SEM analyses of the entire sample.

**Table 1:** Results of measurement invariance tests by age-group.

	*χ* ^2^	*df*	CFI	TLI	RMSEA (90% CI)	SRMR	△CFI	Comparison
1. Configural invariance	1354.16	547	0.94	0.93	0.06 (0.05–0.06)	0.06		
2. Metric invariance	1376.21	592	0.94	0.93	0.06 (0.05–0.06)	0.06	<0.01	1 and 2
3. Scalar invariance	1445.97	610	0.94	0.93	0.06 (0.05–0.06)	0.06	<0.01	2 and 3

90% CI = 90% confidence interval.

## Results

### Descriptive statistics

We present means with standard deviations and Pearson correlations for all research variables in Table 2, and differences between younger and older adults in all variables in Table 3.

**Table 2:** The Pearson correlations and descriptive statistics for all research variables.

	1	2	3	4	5	6	7	8	9
1. Spouse support	–								
2. Child support	0.34**	–							
3. Friend support	0.24**	0.36**	–						
4. Parent support	0.27**	0.47**	0.39**	–					
5. Autonomy	0.16**	−0.01	0.02**	−0.04**	–				
6. Competence	0.14**	0.16**	0.17**	0.12*	0.44**	–			
7. Relatedness	0.16**	0.21**	0.30**	0.18**	0.38**	0.73**	–		
8. Happiness	0.25**	0.25**	0.24**	0.22**	0.52**	0.62**	0.60**	–	
9. Depressive symptoms	−0.29**	−0.08	−0.09*	−0.09*	−0.66**	−0.41**	−0.41**	−0.66**	–
*M*	3.56	2.84	3.06	2.91	3.80	3.31	3.35	3.22	15.90
*SD*	0.95	0.92	0.84	1.00	0.71	0.82	0.81	0.54	10.34
Skewness	−0.69	0.13	−0.27	−0.13	−0.39	−0.41	−0.41	0.04	0.70
Kurtosis	0.07	−0.55	−0.20	−0.73	−0.28	0.21	0.20	0.46	−0.36

*n* = 807; Demographic information (i.e., age, gender, income, and education) was adjusted in the model; * *p* < 0.05; ** *p* < 0.01.

**Table 3:** Age-group differences on all research variables.

Variables	Younger adults (*n* = 605)		Older adults (*n* = 202)		*t*	Cohen’s *d*
	*M*	*SD*	*M*	*SD*		
Social Support						
Spouse	3.57	0.96	3.55	0.93	−0.19	0.02
Child	2.74	0.90	3.04	0.93	3.66***	0.32
Friend	3.08	0.85	3.00	0.82	−1.19	0.05
Parent	2.98	0.96	2.68	1.07	−3.56***	0.30
Satisfaction of needs						
Autonomy	3.74	0.72	4.00	0.64	4.90***	0.39
Competence	3.28	0.81	3.41	0.85	1.86	0.15
Relatedness	3.32	0.82	3.43	0.82	1.74	0.15
Psychological well-being						
Happiness	3.19	0.54	3.31	0.52	2.94**	0.23
Depressive symptoms	16.85	10.55	13.04	9.11	−4.94**	0.39

Younger adults = 30–59 years, older adults = 60–69 years; * *p* < 0.05; ** *p* < 0.01; *** *p* < 0.001.

Overall, younger adults reported greater parental support (*t* = −3.56, *p* < 0.001, *d* = 0.30) and higher depressive symptoms (*t* = −4.94, *p* < 0.01, *d* = 0.39) than did older adults, whereas older adults reported greater support by a child than did younger adults (*t* = 3.66, *p* < 0.001, *d* = 0.32). In addition, older adults reported greater satisfaction of the need for autonomy (*t* = 4.90, *p* < 0.001, *d* = 0.39) and happiness (*t* = 2.94, *p* < 0.01, *d* = 0.23).

### Main SEM analyses

**Figure 1:**
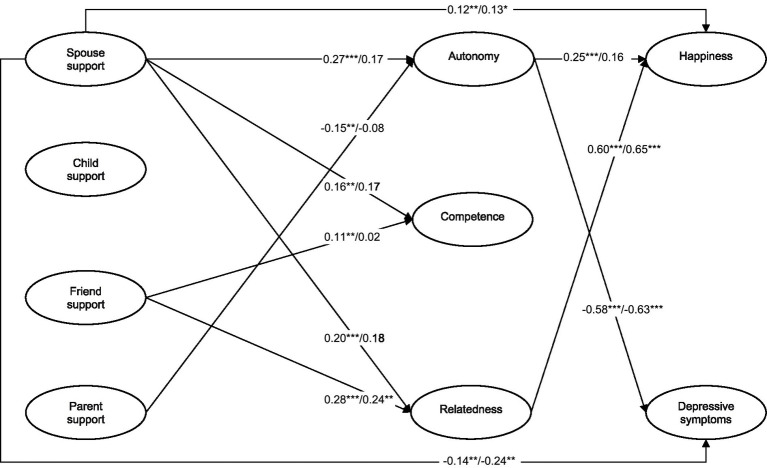
The final structural paths on the associations between social support, satisfaction of needs, well-being. The path coefficients before and after the slash are for younger and older adults, respectively; *n* = 807; * *p* < 0.05. ** *p* < 0.01. *** *p* < 0.001.

To investigate our research hypotheses, we estimated a structural equation model representing the direct effects of social support from different relationship types on happiness and depressive symptoms, and indirect effects through satisfaction of the needs for autonomy, competence, and relatedness. Mediating the effects of social support from different relationship types, satisfaction of the needs for autonomy, competence, and relatedness was hypothesized to affect happiness and depressive symptoms. Because the three needs and the two well-being indicators are interrelated, the three latent factors of the needs and the two latent factors of well-being were allowed to covary. Also, we conducted a multi-group comparison between younger and older adults to investigate the invariance of the structural equation model (see footnote 2). The final SEM model was a good fit to the data [*χ*^2^/*df* = 2.32, *p* < 0.001, CFI = 0.94, TLI = 0.93, RMSEA = 0.05 (90% CI of the RMSEA = 0.05–0.06), and SRMR = 0.07], as shown in Figure 1. Overall, social support from different relationship types and satisfaction of the three needs explained 65 and 44% of the variance in happiness and depressive symptoms, respectively. Below, we provide our main findings on the direct effects of social support on happiness and depressive symptoms, the mediating effects of satisfaction of the three needs, and a group comparison between younger and older adults based on our research hypotheses.

### The direct effects of support on happiness and depressive symptoms (hypothesis 1)

The direct path coefficients from spousal support to happiness (β = 0.21, *p* < 0.001) and depressive symptoms (β = −0.31, *p* < 0.001), friend support to happiness (β = 0.12, *p* < 0.01) in the absence of mediators were significant. The direct-effect model fitted the data well [*χ*^2^/*df* = 3.78, *p* < 0.001, CFI = 0.96, TLI = 0.95, RMSEA = 0.06 (90% CI of the RMSEA = 0.05–0.06), and SRMR = 0.06]. Thus, our first hypothesis was supported.

### The mediating effects of satisfaction of the needs (hypothesis 2)

**Figure 2:**
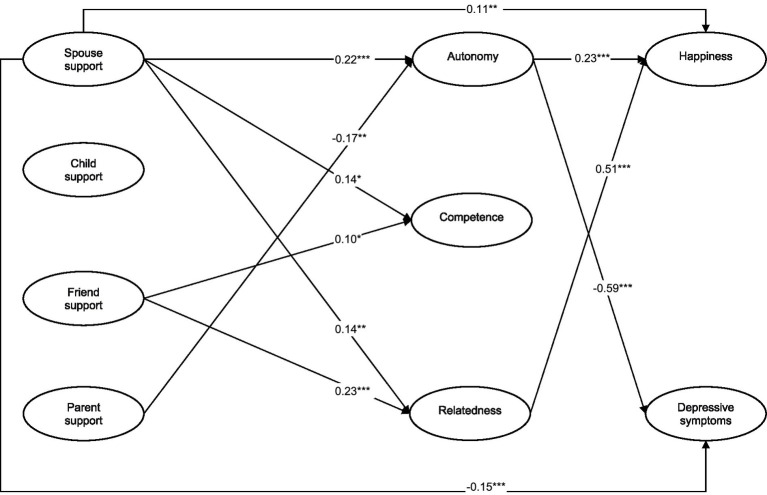
The mediating effects of satisfaction of needs on the associations between social support and well-being. *n* = 807; * *p* < 0.05. ** *p* < 0.01. *** *p* < 0.001.

Figure 2 presents the results for the mediation model linking social support from different relationship types to happiness and depressive symptoms with satisfaction of the three needs as mediators. We found that spousal support had a significant direct effect on satisfaction of the needs for autonomy (β = 0.22, *p* < 0.001), competence (β = 0.14, *p* < 0.05), and relatedness (β = 0.14, *p* < 0.01), and had a significant indirect effect on happiness and depressive symptoms, mediated by satisfaction of the needs for autonomy and relatedness. Also, friend support had a significant direct effect on satisfaction of the needs for competence (β = 0.10, *p* < 0.05) and relatedness (β = 0.23, *p* < 0.001), and had a significant indirect effect on happiness, mediated by satisfaction of the need for relatedness. The indirect-effect model fitted the data well [*χ*^2^/*df* = 3.25, *p* < 0.001, CFI = 0.95, TLI = 0.94, RMSEA = 0.05 (90% CI of the RMSEA = 0.05–0.06), and SRMR = 0.06].

To specifically examine the mediating effects of satisfaction of needs, we conducted a bootstrapping procedure (Shrout and Bolger, 2002). If the 95% of confidence interval for the mediating-effect estimates does not include zero, the mediating effects are significant at the 0.05 level (Hayes and Scharkow, 2013). Results in Table 4 indicate that the 95% CI for the mediating effects of satisfying the needs for autonomy and relatedness did not include zero, indicating that satisfaction of the needs for autonomy and relatedness significantly mediated the associations between social support from spouses and friends and happiness and depressive symptoms. The bootstrap tests demonstrated that the mediating effect of satisfying the needs for autonomy and relatedness accounted for 5 and 14% of the total effect, respectively.

**Table 4:** Bootstrap analyses of the magnitude and statistical significance of indirect effects.

Path			β	*SE*	*z*	95% CI	
Independent variable	Mediator variable	Dependent variable				Lower	Upper
Spouse support	Autonomy	Happiness	0.05	0.01	2.94	0.02	0.07
		Depressive symptoms	−0.13	0.03	−3.37	−0.15	−0.04
	Relatedness	Happiness	0.07	0.02	2.27	0.01	0.10
Friend support	Relatedness	Happiness	0.12	0.03	3.38	0.06	0.19

z = z-score; 95% CI = 95% confidence interval.

To compare the strength of different mediating effects, the indirect effects of social support from spouses and friends on happiness and depressive symptoms were calculated separately for the mediating effects of satisfaction of the needs for autonomy and relatedness. We found that the indirect effect of spousal support on depressive symptoms *via* satisfaction of the need for autonomy (β = −0.13, 95% CI = [−0.15 – −0.04]) was the strongest, followed by the indirect effect of friend support on happiness *via* satisfaction of the need for relatedness (β = 0.12, 95% CI = [0.06–0.19]). The indirect effect of spousal support on happiness *via* satisfaction of the need for autonomy (β = 0.05, 95% CI = [0.02–0.07]) was the weakest. Thus, our hypothesis 2 was partially supported.

### Equivalence by age groups (hypothesis 3)

As presented in Figure 1, we conducted a multigroup SEM analysis to find out if the structural equation model was equivalent between younger and older adults (see footnote 1). We compared two nested models: (a) one in which the measurement model is equivalent, but structural paths are free to differ by age groups, and (b) one in which the measurement model and structural paths are equivalent by age groups. The structural equation model without equivalence constraints showed a better fit to the model (Δ*χ*^2^ = 5.54, *p* > 0.05, ΔCFI < 0.001), which indicates that the structural path coefficients were significantly different between younger and older adults.

The structural paths from spousal and friend support to satisfaction of the needs for autonomy, competence, and relatedness was significantly different between younger and older adults.^3^ The path coefficients from spousal support to satisfaction of the needs for autonomy (β = 0.27, *p* < 0.001), competence (β = 0.16, *p* < 0.01), and relatedness (β = 0.20, *p* < 0.001), and from friend support to satisfaction of the need for competence (β = 0.11, *p* < 0.01) were positively significant for younger adults, whereas such paths were not significant for older adults. Also, the path coefficient from friend support to satisfaction of the need for relatedness was greater for younger adults (β = 0.28, *p* < 0.001) than for older adults (β = 0.24, *p* < 0.01). In addition, the structural paths from satisfaction of the needs for autonomy and relatedness to happiness and depressive symptoms were significantly different between younger and older adults. The path coefficient from satisfaction of the need for autonomy to happiness was positively significant for younger adults (β = 0.25, *p* < 0.001), whereas that path was not significant for older adults. However, the path coefficients from satisfaction of the need for autonomy to depressive symptoms and from satisfaction of the need for relatedness to happiness was comparable between older adults (β = −0.63, *p* < 0.001; β = 0.65, *p* < 0.001) and younger adults (β = −0.58, *p* < 0.001; β = 0.60, *p* < 0.001). Thus, our hypothesis 3 was not supported.

## Discussion

The aim of this study was to investigate how social support from different relationship types relates to younger and older adults’ happiness and depressive symptoms, and whether the satisfaction of individual needs for autonomy, competence, and relatedness mediates the link between social support and well-being. Our focus was on how important relationship-specific social support and individual satisfaction of needs were for happiness and depressive symptoms, and on the age-group differences in the associations between social support, satisfaction of needs, and well-being. The findings lend support to our approach of distinguishing different relationship types and the three needs. Our results underscored that social support from spouses and friends, and the satisfaction of the needs for autonomy and relatedness, had the strongest effects on happiness and depressive symptoms, and that the beneficial effects of social support and satisfaction of needs differed between younger and older adults.

### Social support from different relationship types and psychological well-being

As anticipated, social support received from spouses, children, friends, and parents had different effects on happiness and depressive symptoms, and social support from one’s spouse and friends had the most robust influence on greater happiness and decreased depressive symptoms. This finding is consistent with prior studies that emphasize the significance of spouses and friends as sources of social support for psychological well-being (Fiori et al., 2006; Birditt and Antonucci, 2007). A few studies that considered multiple types of social support for Japanese (Okabayashi et al., 2004) and American (Chen and Feeley, 2014) older adults showed that social support by spouses, family, and friends has different effects, and spousal support has particularly great influence on well-being. It is noteworthy that our findings provide additional empirical validation for the salient roles of spousal support in promoting happiness and alleviating depressive symptoms based on South Korean younger and older adults. Our findings along with those from existing research suggest that social support from multiple relationship types can be associated with psychological well-being in different ways and underscore the importance of considering differentiated social relationships.

### The mediating effects of satisfaction of basic psychological needs

Based on self-determination theory, we anticipated that the satisfaction of the needs for autonomy, competence, and relatedness mediates the link between social support and happiness and depressive symptoms. However, our hypothesis was only partially supported, because spousal and friend support were associated with happiness and depressive symptoms only *via* satisfaction of the needs for autonomy and relatedness. Our finding does not indicate that the satisfaction of the need for competence does not play a role; rather, the salience of competence need satisfaction in mediating effects was weaker than for that of the other two needs.^4^ This is consistent with the proposition of SDT, which highlights that the fulfillment of the need for autonomy is a prerequisite for the satisfaction of the needs for competence and relatedness, because satisfaction of the need for autonomy provides individuals with psychological freedom to pursue satisfaction of the needs for competence and relatedness (Deci and Ryan, 2014).

Focusing on satisfaction of each of the three specific needs, the associations from social support to well-being *via* satisfaction of the needs were generally replicated (Vermote et al., 2022). Thus, this study corroborates prior research on satisfaction of basic psychological needs as mediating the link between social support and well-being. However, we made an important addition to the literature on the independent and additive effects of each specific need on well-being. Whereas most studies have often aggregated satisfaction of each need into a global measure, we distinguished satisfaction of each specific need as distinct variables rather than as composites. Our results for simultaneously testing satisfaction of the three needs showed that satisfaction of the needs for autonomy and relatedness exerted additive contributions to happiness and depressive symptoms. Hence satisfaction of all three needs is not just crucial for individuals’ optimal functioning (Ryan and Deci, 2017), but their effects could be additive, with each need having crucial effects on psychological well-being (Reis et al., 2000).

### Age-group differences in the links between support, need satisfaction, and well-being

Based on life span theories, we anticipated that the associations between social support from different relationship types, satisfaction of the three needs, and happiness and depressive symptoms could differ between younger and older adults. Overall, the beneficial effects of social support from spouses and friends on satisfaction of the three needs were greater for younger adults than for older adults. However, friend support was associated with satisfaction of the need for relatedness about equally in both groups, and the direct effects from one’s spouse on happiness and depressive symptoms were greater for older adults than for younger adults. These results are in line with prior evidence on socioemotional selectivity theory (Carstensen, 1995), which emphasizes that focal relationships and their salience can change over the life course. Compared to younger adults who envision unlimited time for themselves, older adults would envision less amount of time left to live and would thus focus on those who are the most important to them. Accordingly, social support from family members, such as spouses, become more critically tied to their psychological well-being (Segrin, 2003). The direct (not *via* satisfaction of needs) effects of emotional or instrumental support from one’s spouse (e.g., companionship, and assistance with daily living) on well-being would become particularly critical as their physical health begins to decline (Cantor, 1979; Cornwell and Waite, 2009).

The strong effect of friend support on relatedness satisfaction for both younger and older adults is also noteworthy. In addition to the prior research that has focused on the effect of harmonious and supportive interactions with family members (Solomon and Jackson, 2014), considerable attention also has been paid to non-familial relationships, such as friends. For younger adults, social support from friends can have salient influence in reinforcing their self-esteem and reducing loneliness (Arroyo et al., 2022). Our findings further underscore that friends can also be the major and preferred source of support for older adults, because they can confide in friends about the aging processes (Walen and Lachman, 2000). Friend support could play particularly significant roles in psychological well-being in later life as familial support generally declines due to death of a spouse and direct family members (Gupta and Korte, 1994).

Our findings demonstrated that the positive association between satisfaction of the need for relatedness and happiness, and the negative association between satisfaction of the need for autonomy and depressive symptoms, were comparable between younger and older adults. The similar associations between satisfaction of needs and well-being between younger and older adults indicate that the effects of satisfaction of needs on well-being is universal for individuals in all life stages as proposed by SDT (the universality assumption; Ryan and La Guardia, 2000). Whereas this universality assumption was overall supported, the positive association between satisfaction of the need for autonomy and happiness was significant only for younger adults. This finding was unexpected. A potential explanation may be that in line with the selective optimization with compensation assumption, aging requires individuals to prioritize new goals and narrow down previous perspectives (Baltes and Baltes, 1990; Carstensen, 2006; Freund, 2017). Accordingly, older adults may gradually accept that their autonomy is diminished by aging-related losses in behavioral and psychological functioning and therefore may rely on the satisfaction of other needs, such as relatedness, to maintain better psychological well-being. In line with this reasoning, Neubauer et al. (2017) suggested that individuals need to downplay psychological needs that cannot be fulfilled in a certain life stage and should focus on needs that can be satisfied more easily. Given that research that considers both younger and older adults simultaneously and investigates age-group differences is rare, future research should use a different sample to examine this issue in more detail and replicate our findings.

### Limitations and future research

The current study has several limitations that should be addressed in future research. First, all of the variables that we used in this work were based on self-reported measures. Because relying on self-reported information could affect the magnitude of the associations between constructs, future research should obtain information from multiple sources to improve the validity of the research constructs. Second, this study was based on a cross-sectional dataset. Thus, causal inferences should be interpreted with caution. Future research that incorporates a longitudinal dataset could build on our results to establish the directionality of the associations.

## Conclusion

Despite these limitations, this study provides a novel contribution to the literature, in that it elucidates how relationship-specific social support is differently associated with both positive and negative indicators of psychological well-being, and whether satisfaction of the three basic psychological needs mediate these associations. Our findings also respond to the gap in the literature about age-group differences in the links between social support, satisfaction of needs, and well-being by simultaneously examining younger and older adults. Our findings underscore that spousal and friend support becomes more critical for psychological well-being over the course of life, and the satisfaction of the needs for autonomy and relatedness is consequential for greater happiness and decreased depressive symptoms as people age. Collectively, our results accentuate that distinguishing different relationships and satisfaction of needs in tandem with comparing varying age groups are important to expand our understanding of the role of social support in psychological well-being across the adult lifespan.

## Data availability statement

The original contributions presented in the study are included in the article/Supplementary material, further inquiries can be directed to the corresponding author.

## Ethics statement

The studies involving human participants were reviewed and approved by the Human Subjects Ethics committee of the Jeonbuk National University’s Institutional Review Board. The patients/participants provided their written informed consent to participate in this study.

## Author contributions

HS conceived of the study, helped the analyses and interpretation of the data, and drafted the manuscript. CP did the analyses and interpreted the data. All authors contributed to the article and approved the submitted version.

## Funding

The research received funding from the Brain Korea 21 fourth project of the Korea Research Foundation (Jeonbuk National University, Psychology Department no. 4199990714213).

## Conflict of interest

The authors declare that the research was conducted in the absence of any commercial or financial relationships that could be construed as a potential conflict of interest.

## Publisher’s note

All claims expressed in this article are solely those of the authors and do not necessarily represent those of their affiliated organizations, or those of the publisher, the editors and the reviewers. Any product that may be evaluated in this article, or claim that may be made by its manufacturer, is not guaranteed or endorsed by the publisher.

## Supplementary material

The Supplementary material for this article can be found online at: https://www.frontiersin.org/articles/10.3389/fpsyg.2022.1051968/full#supplementary-material

## Footnotes

^1^We recruited participants from an online research participant system, which has a nationally representative sample aged 14 years and older. We used stratified probability sampling to obtain representative samples of different age groups of women and men in adulthood. We invited the pool of participants to take part in the study by posting an information describing the research and a survey link on the message board. The current dataset was part of a larger project, and we did not have particular inclusion or exclusion criteria other than their ages. The final sample was 1,033 adults (50.1% females, 100% South Korean) aged between 20 and 69 years (*M* = 44.05 years, *SD* = 13.71) who resided in South Korea. Each decade of life span (i.e., 20–29 years, *n* = 210; 30–39 years, *n* = 203; 40–49 years, *n* = 204; 50–59 years, *n* = 209; 60–69 years, *n* = 207) and the number of participants by geographical region (i.e., 5–7% for 17 different municipalities) were well represented. Because this study involves the spousal relationship and there were very few adults who were married in their 20s, we only used the participants who were aged 30 years and older. Excluding 16 outliers, the final sample included 807 adults (50.40% male) aged between 30 and 69 years. We provided detailed procedural information in the Appendix as a flow chart.

^2^We analyzed preliminary models for different age groups in their 30s, 40s, 50s, and 60s to find out if the associations between social support from different relationship types, satisfaction of needs, and well-being differ between age groups. As anticipated, results were comparable between the 30s through the 50s, and the main differences were found between 30s–50s and 60s. Thus, we conducted a group comparison between younger (30s–50s) and older (60s) adults to investigate the invariance of the structural equation model.

^3^We analyzed our models for different age groups in their 30s, 40s, 50s, and 60s to check if sample sizes affected the results of the associations. However, as in the preliminary analyses, results were generally comparable between the 30s through the 50s, and the main differences were found between 30s–50s and 60s.

^4^We analyzed our models for each individual need to see if satisfaction of the needs for autonomy, competence, and relatedness mediates the link between social support from different relationship types and well-being when they are not simultaneously considered. As seen in the Appendix, all three needs did mediate the link between social support and well-being when they were investigated individually.
