# Supplementary material for: Social support and psychological well-being in younger and older adults: The mediating effects of basic psychological need satisfaction
Source: Front Psychol. 2022 Nov 25;13:1051968. doi: 10.3389/fpsyg.2022.1051968 (PMC9733472; doi:10.3389/fpsyg.2022.1051968)
Supplement: Supplementary file 1 [file Data_Sheet_1.docx]

**Appendix**

Online research participant system:

a nationally representative sample aged 14 years and older

20s excluded:

9 married participants

(*n* = 210)

20s – 60s South Korean adults:

stratified probability sampling by different age groups of women and men

(*n* = 1,033)

Data collection

30s – 60s South Korean adults:

575 participants who were married

(*n* = 823)

Outliers excluded

(*n* = 16)

30s – 60s South Korean adults

(*n* = 807)

Screening

Final data

Figure A.1. A flow chart on procedural information

Table A.1 Demographic information on the participants

|  | 30s | 40s | 50s | 60s | All |
| --- | --- | --- | --- | --- | --- |
|  |  |  |  |  |  |
| Sample size (*n*) | 203 | 204 | 209 | 207 | 823 |
| Age, *mean (SD)* | 34.51 (2.75) | 43.97 (2.91) | 53.20 (2.70) | 63.27 (2.73) | 48.83 (11.02) |
| Gender |  |  |  |  |  |
| Men, *n* (*%*) | 100 (24.10%) | 104 (25.06%) | 105 (25.30%) | 106 (25.54%) | 415 |
| Women, *n* (*%*) | 103 (25.25%) | 100 (24.51%) | 104 (25.49%) | 101 (24.75%) | 408 |
| Education |  |  |  |  |  |
| <High school, *n* (*%*) | 21 (12.65%) | 37 (22.29%) | 47 (28.31%) | 61 (36.75%) | 166 |
| Some college, *n* (*%*) | 161 (28.60%) | 149 (26.47%) | 133 (23.62%) | 120 (21.31%) | 563 |
| Graduate school, *n* (*%*) | 21 (22.34%) | 18 (19.15%) | 29 (30.85%) | 26 (27.66%) | 94 |
| Retirement status |  |  |  |  |  |
| Retired, *n* (%) | 3 (1.82%) | 11 (6.67%) | 26 (15.76%) | 125 (75.76%) | 165 |
| Not retired, *n* (%) | 200 (30.40%) | 193 (29.33%) | 183 (27.81%) | 82 (12.46%) | 658 |
| Income |  |  |  |  |  |
| <$10,000, *N* (*%*) | 24 (14.55%) | 33 (20.00%) | 56 (33.94%) | 52 (31.52%) | 165 |
| $10,000-$20,000, *n* (*%*) | 11 (16.92%) | 15 (23.08%) | 14 (21.54%) | 25 (38.46%) | 65 |
| $20,000-$30,000, *n* (*%*) | 30 (27.52%) | 24 (22.02%) | 27 (24.77%) | 28 (25.69%) | 109 |
| $30,000-$40,000, *n* (*%*) | 45 (32.61%) | 34 (24.64%) | 21 (15.22%) | 38 (27.54%) | 138 |
| >$40,000, *n* (*%*) | 93 (26.88%) | 98 (28.32%) | 91 (26.30%) | 64 (18.50%) | 346 |
| Marital status |  |  |  |  |  |
| Single, *n* (*%*) | 111 (61.33%) | 49 (27.07%) | 16 (8.84%) | 5 (2.76%) | 181 |
| Married, *n* (*%*) | 85 (14.99%) | 144 (25.40%) | 168 (29.63%) | 170 (29.98%) | 567 |
| Divorced, *n* (*%*) | 3 (6.82%) | 7 (15.91%) | 16 (36.36%) | 18 (40.91%) | 44 |
| Bereavement, *n* (*%*) | 0 (0.00%) | 1 (6.67%) | 5 (33.33%) | 9 (60.00%) | 15 |
| Child |  |  |  |  |  |
| One or more, *n* (*%*) | 54 (9.41%) | 142 (24.74%) | 183 (31.88%) | 195 (33.97%) | 574 |
| None, *n* (*%*) | 149 (59.84%) | 62 (24.90%) | 26 (10.44%) | 12 (4.82%) | 249 |
| Children live together |  |  |  |  |  |
| Yes, *n* (%) | 54 (12.13%) | 136 (30.56%) | 150 (33.71%) | 105 (23.60%) | 445 |
| No, *n* (%) | 0 (0.00%) | 6 (4.65%) | 33 (25.58%) | 90 (69.77%) | 129 |
|  |  |  |  |  |  |

0.54***

0.21***

-0.60***

-0.16**

-0.16***

0.19***

0.17***

Figure A.2. The structural paths on the associations between social support, satisfaction of autonomy, and well-being.

*Note*. *n* = 807; * *p* < 0.05. ** *p* < 0.01. *** *p* < 0.001.

0.64***

0.13*

-0.32***

-0.10*

0.15***

-0.20***

0.13***

Figure A.3. The structural paths on the associations between social support, satisfaction of competence, and well-being.

*Note*. *n* = 807; * *p* < 0.05. ** *p* < 0.01. *** *p* < 0.001.

0.68***

0.13*

-0.34***

0.25***

0.14***

-0.18***

Figure A.4. The structural paths on the associations between social support, satisfaction of relatedness, and well-being

*Note*. *n* = 807; * *p* < 0.05. ** *p* < 0.01. *** *p* < 0.001.

\
